# A Lung Cavity Opened Surgically

**Published:** 1889-05-18

**Authors:** 


### A LUNG CAVITY OPENED SURGICALLY.

It often happens that a diseased process kills a patient, because it is so situated that it cannot be "got at." Until recently the best surgeons were afraid, for example, to make openings into cavities in the lungs, even though they were sometimes convinced that the free discharge of matter from such cavities was all that was required to effect a cure. An interesting case is reported from the Devon and Exeter Hospital, which convincingly proves the value of bold surgical treatment when such measures are available. In the winter of 1887 a gentleman, thirty-three years of age, sought medical advice under a complication of troubles involving the kidneys, and giving rise to abscesses which opened in the back. He improved for a time under treatment, and seemed to be getting quite well; but just when hope was at its highest he suddenly grew worse, and developed symptoms of inflammation of the lungs. At first this new complication seemed disposed to run a favourable course; but after a time the breathing became suffocatingly difficult, the cough was incessant, profuse night-sweats set in, and there was every sign of the most imminent danger to life. For weeks this continued, until the patient was reduced to a skeleton, and the odour of his breath was so offensive as to be poisonous to himself and injurious to his attendants. A careful examination by the medical men in charge left no doubt that there was a cavity in the posterior portion of one of the lungs, and that the matter which was constantly being formed in that cavity gave rise to the fever, the cough, the offensive breath, the loss of flesh, and all the other alarming and dangerous symptoms. Now there is no "drug" treatment known to medical science which will promptly and effectively remove, or prevent the formation of, pus in such circumstances. And yet the pus is killing the patient. Reason says it must be removed at all risks. If it be not removed the patient will die. If it be removed he may live, and at most he can but die. On the 2nd of July, after weeks of fruitless treatment, it was explained to the patient that his one chance of life lay in a surgical operation. He readily consented, and without the use of an anæsthetic the skin was cut through, and a hollow "aspirating" needle pushed three or four inches into the cavity of the lung. As had been anticipated, a free flow of matter followed the introduction of the needle. The surgeons then proceeded to follow the needle with the knife, and an opening was made into the cavity sufficient to admit of the passage of a drainage tube. This was fixed in place, and for days there was a profuse discharge of pus through the tube. The result of the treatment was that the alarming symptoms began to abate immediately. In less than two months the cough was quite gone, the strength had returned greatly, and convalescence was established. Gradually the patient gained 28lb. in weight: and before the end of the year he was able to return to his work. But forthe surgical operation he must certainly have died.

